# Differential Replication and Cytokine Response between Vaccine and Very Virulent Marek’s Disease Viruses in Spleens and Bursas during Latency and Reactivation

**DOI:** 10.3390/v15010006

**Published:** 2022-12-20

**Authors:** Bo Jiang, Jing Wang, Mengyao Cao, Huan Jin, Wenxiao Liu, Jing Cheng, Linyi Zhou, Jian Xu, Yongqing Li

**Affiliations:** 1 Institute of Animal Husbandry and Veterinary Medicine, Beijing Academy of Agricultural and Forestry Sciences, Beijing 100097, China; 2 College of Veterinary Medicine, China Agricultural University, Beijing 100193, China; 3 College of Animal Science and Technology, Beijing University of Agriculture, Beijing 102206, China; 4 Lanzhou Veterinary Research Institute, Chinese Academy of Agricultural Sciences, Lanzhou 730070, China

**Keywords:** Marek’s disease virus, lymphoid organs, virus duplication, cytokines

## Abstract

Marek’s disease virus (MDV) infection results in Marek’s disease (MD) in chickens, a lymphoproliferative and oncogenic deadly disease, leading to severe economic losses. The spleen and bursa are the most important lymphoid and major target organs for MDV replication. The immune response elicited by MDV replication in the spleen and bursa is critical for the formation of latent MDV infection and reactivation. However, the mechanism of the host immune response induced by MDV in these key lymphoid organs during the latent and reactivation infection phases is not well understood. In the study, we focused on the replication dynamics of a vaccine MDV strain MDV/CVI988 and a very virulent MDV strain MDV/RB1B in the spleen and bursa in the latent and reactivation infection phases (7–28 days post-inoculation [dpi]), as well as the expression of some previously characterized immune-related molecules. The results showed that the replication ability of MDV/RB1B was significantly stronger than that of MDV/CVI988 within 28 days post-infection, and the replication levels of both MDV strains in the spleen were significantly higher than those in the bursa. During the latent and reactivation phase of MDV infection (7–28 dpi), the transcriptional upregulation of chicken IL-1β, IL6, IL-8L1 IFN-γ and PML in the spleen and bursa induced by MDV/RB1B infection was overall stronger than that of MDV/CVI988. However, compared to MDV/RB1Binfection, MDV/CVI988 infection resulted in a more effective transcriptional activation of CCL4 in the latent infection phase (7–14 dpi), which may be a characteristic distinguishing MDV vaccine strain from the very virulent strain.

## 1. Introduction

Marek’s disease (MD), caused by Marek’s disease virus (MDV) in chickens, is a lymphoproliferative, oncogenic disease [1]. MDV infection leads to oncogenic immunosuppression, which seriously affects the production performance of the poultry industry and results in huge economic losses [2]. MDV replicates in lymphoid tissues, resulting in lymphocytosis, which shows many considerable biological similarities with lymphoid neoplasms related to the Epstein-Barr virus and contributes substantially to the understanding of herpes virus-associated oncogenicity [3]. MDV infection is caused by the inhalation of mature virions attached to dust and dander, which are continuously shed from the feather follicle epithelium of infected chickens. After reaching the respiratory epithelium, macrophages transport MDV to the bursa, spleen, and other lymphoid organs for replication [4]. During the early cytolytic stages of MDV infection (2–7 days post-inoculation [dpi]), B lymphocytes are primarily targeted by infected cells, which subsequently infect activated CD4 T lymphocytes. The latent infection phase occurs at 7–10 dpi, and lymphocytes harboring the MDV genome show restricted expression of some latent-related genes. At approximately 14–21 dpi, the reactivation of MDV in CD4+ T cells initiates a late phase of cytolysis and immunosuppression, and thus, the second cytolytic infection occurs. After 28 dpi, lymphocytes infected with MDV migrate to the visceral organs and peripheral nerves to generate T-cell lymphomas and immunosuppression, resulting in blindness, paralysis, and death [5]. Thus, lymphocytes are the main target cells for viral replication and systemic spread within the host during early and latent MDV infections [6].

Over the last few decades, MDV has progressively become more pathogenic, ranging from mild to virulent (v), very virulent (vv), and very virulent+ (vv+) [7]. As a result, this has necessitated the introduction of increasingly effective vaccine strains to prevent MD outbreaks with the increasing virulence of wild MDV [8]. CVI988/Rispens is one of the most effective commercially available MDV vaccines, which can induce lifelong immune protection in chickens [9]. Moreover, the recombinant MDV CVI988 prepared by bacterial artificial chromosome technology has the characteristics of efficient replication and strong immune protection, which offers MDV/CVI988 high commercial value for the development of a virus vaccine vector [10]. As a very virulent strain, MDV/RB1B can cause MD in susceptible chickens, but not in those that are MDV/CVI988-vaccinated [11,12]. Notably, although vaccination protects against MD after infection with virulent MDV strains, vaccine-induced immunity fails to induce sterilizing immunity to eliminate the virus, resulting in its further spread via feather and dander [5]. This may be due to MDV Meq oncoprotein amino acid mutations, which alter the virulence of the virus, as reported by Conradie et al., and are associated with allowing the virus to overcome vaccinal protection [13]. On the other hand, the intense invasiveness and virulence of vvMDV makes it possible to break through the protection of vaccine-induced immunity [14]. However, studies on how attenuated MDV vaccines activate host-protective immune responses against vvMDV strains are still scarce.

Our previous study reported that MDV/CVI988 and MDV/RB1B (a vvMDV strain) elicited different host immune responses in early cytolytic infection phase (5 dpi) in vivo experiments. In particular, the transcription level of some cytokines in the splenic lymphocytes, including IL-1β, IL-6, IL-8L1, and CCL4, which are related to B cell activation and antigenic signal transduction to T cells, were significantly upregulated by MDV/CVI988 infection compared with MDV/RB1B infection [15]. Considering the different pathological responses of the host after infection with the MDV vaccine strain and virulent strain, we speculate that the replication and immune activation status of MDV with different virulence levels also differ during the latent infection and reactivation phases. Here, we investigated the viral replication curves of the vaccine strain and vvMDV strain in the host spleen and bursa during the latent and reactivation phases. In addition, the mRNA levels of interferons and cytokines related to immune activation and inflammatory responses in chickens within 28 dpi with the vaccine MDV strain CVI988 and very virulent MDV strain RB1B were detected and analyzed for the first time. Our results demonstrated that the replication ability of the vvMDV strain was stronger than that of the vaccine strain, and the cytokine responses induced by different strains were positively associated with the immune status and the establishment of latent infection by the virulent strain.

## 2. Materials and Methods

### 2.1. Ethics Statement

We carried out the animal experiments in strict accordance with the requirements of the Laboratory Animal Requirements of Environment and Housing Facilities (GB14925-2010, National Laboratory Animal Standardization Technical Committee) and the Chinese Regulations of Laboratory Animals Guidelines (Ministry of Science and Technology of People’s Republic of China). The experimental procedures were approved and audited by Beijing Academy of Agricultural and Forestry Sciences animal welfare committee (15 December 2017, license number 2017-0039, ID: SYXK(Jing)).

### 2.2. Viruses, Experimental Animals, and Design

Very virulent MDV strain MDV/RB1B (passage 10) was preserved in our laboratory. MDV vaccine strain MDV/CVI988 (passage 45) was purchased from Linyv Avian Health Co., Ltd., Beijing, China. Animal experiments were conducted in specific pathogen-free (SPF) chicken isolators in a temperature-controlled animal house. The SPF chickens (White Leghorn lines) used in this study were purchased from Beijing Boehringer Ingelheim Vitao Biotechnology Co., Ltd., Beijing, China. Seventy-two one-day-old SPF chickens (mixed-sex) were randomly divided into 3 groups, and each group (24 chickens) was separately reared. The MDV-infected chickens were subcutaneously injected in the neck with 5000 plaque-forming units (pfu) of MDV/RB1B or MDV/CVI988 in 0.2 mL. The mock-treated chickens in the control group were injected with 105 chicken embryo fibroblast cell (0.2 mL) in the same way. Bursas and spleens were sampled from the euthanized chickens of 3 experimental groups (control, MDV/CVI988, MDV/RB1B) at 7, 14, 21, and 28 dpi. Six chickens were taken from each experimental group at each time point.

### 2.3. RNA and DNA Isolation from Bursa and Spleen

The tissue samples of the bursas and spleens collected from the inoculated and uninoculated chickens were ground after freezing in liquid nitrogen. The total DNA used for quantification using real-time PCR was extracted with 20 mg per sample using the DNeasy Blood & Tissue Kit (Qiagen, The Netherland), and each sample was eluted with 40 µL of deionized H_2_O. Then, 2 µL of each sample DNA was taken for absolute qPCR. The total RNA was extracted with 20mg per sample using the RNeasy Mini Kit (Qiagen, Netherland). Then, cDNA was obtained by reverse transcription using the SuperScript™ III First-Strand Synthesis System (Invitrogen, Waltham, MA, USA, USA), utilizing the RNA as a template. The cDNA concentration of each sample was quantified using the NanoDrop spectrophotometer (ThermoFisher, Waltham, MA, USA) and adjusted to 100 ng/µL. Then, 2 µL of each sample cDNA was taken for a relative qPCR.

### 2.4. Absolute Quantitation of MDV Copies

**Figure 1:**
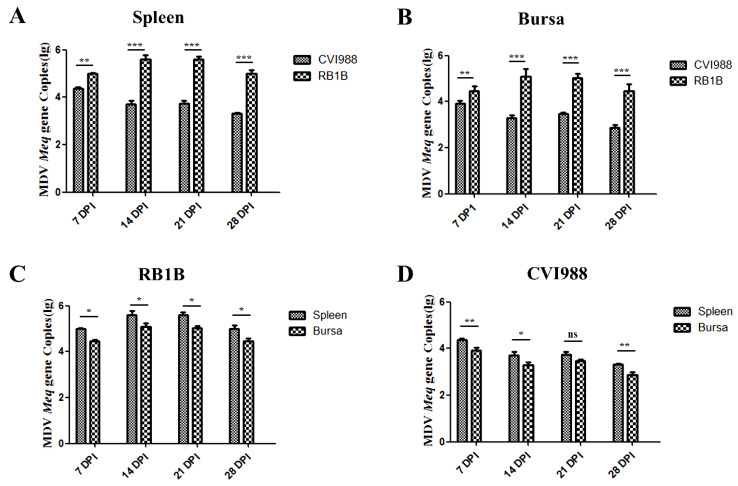
MDV/CVI988 and MDV/RB1B DNA replication in spleen and bursa. (**A**): MDV/CVI988 and MDV/RB1B DNA replication in spleen; (**B**): MDV/CVI988 and MDV/RB1B DNA replication in bursa; (**C**): MDV/RB1B DNA replication in spleen and bursa; (**D**): MDV/CVI988 DNA replication in spleen and bursa. The statistical results are shown as the means ± SD of each experimental group. Statistical significance between experimental groups was assessed by *t*-test (* *p* < 0.05; ** *p* < 0.01; *** *p* < 0.001; ns, no significance).

Serial dilutions ranging from 10^8^ to 10^1^ copies/2 µL of MDV/CVI988-BAC (bacterial artificial chromosome DNA of MDV) [16] were used as standard control samples to quantify the Meq gene copies. The Meq gene primers designed for both MDV/CVI988 and MDV/RB1B were presented in Table 1. The quantitative PCR (qPCR) was performed using CFX96 Real-Time system (Bio-Rad, Hercules, CA, USA) with 20 µL of reaction volume (2 µL of DNA, 0.6 µL of each primer in 10 µM, 10 µL of Bio-Rad iTaq Universal SYBR Green Supermix and 6.8 µL of H_2_O) under the following conditions: pre-incubated at 95 °C for 2 min, and then 40 cycles of 95 °C for 15 s and 58.6 °C for 1 min. The copy number of each standard control and the corresponding CT value were used to establish a standard linear regression curve, and then the copies of the Meq gene were calculated based on the CT value of each sample. The Meq copies presented in Figure 1 are the copies per 1 mg of tissue.

### 2.5. Relative Quantification of Cytokine Expression Levels

The cDNA of the cytokines obtained above was used for the relative qPCR. Each cytokine expression level was normalized by the glyceraldehyde-3-phosphate dehydrogenase (GAPDH) gene. The primer sequences used to detect the target genes and GAPDH are listed in Table 1.The qPCR was also performed using the CFX96 Real-Time system (Bio-Rad, USA) with 20 µL of reaction volume (2 µL of DNA, 0.6 µL of each primer in 10 µM, 10 µL of Bio-Rad iTaq Universal SYBR Green Supermix and 6.8 µL of H_2_O) with the following conditions: initial denaturation at 95 °C for 1 min; 40 cycles of 95 °C for 15 s and 60 °C for 1 min; and finally, a melting curve analysis.

### 2.6. Statistical Analysis

The data were statistically analyzed using SPSS software and GraphPad Prism. The statistical results of the data are presented as mean ± SD in the figures. The significance of the difference between the experimental groups was analyzed by a *t*-test. (***) indicates statistically significant differences (*p* < 0.001); (**) indicates statistically significant differences (*p* < 0.01); (*) indicates statistically significant differences (*p* < 0.05); and (ns) indicates no significant differences.

## 3. Results

### 3.1. Differences in MDV Load between Vaccine and Virulent Strain in Spleen and Bursa

To detect the replication kinetics of the vaccine strain and vvMDV strain during the latent and reactivation infection phases, the DNA of the spleen and bursa of chickens infected with MDV/CVI988 or MDV/RB1B at 7, 14, 21, and 28 dpi were extracted. The MDV copies in the spleen and bursa were detected based on the Meq gene. The results showed that, within 28 dpi, the MDV/RB1B copy number was significantly higher than that of MDV/CVI988 in both the spleen and bursa, indicating that MDV/RB1B was more capable of replication in vivo (Figure 1A,B). Meanwhile, our results signified that both MDV/CVI988 and MDV/RB1B copy numbers in the spleen were higher than that in the bursa (Figure 1C,D), which suggests that the target cell composition and internal environment of the spleen may be more suitable for MDV proliferation during the latent and reactivation infection phases.

### 3.2. Effects of MDV Replication on Interferon and PML Expression in the Spleen and Bursa

**Figure 2:**
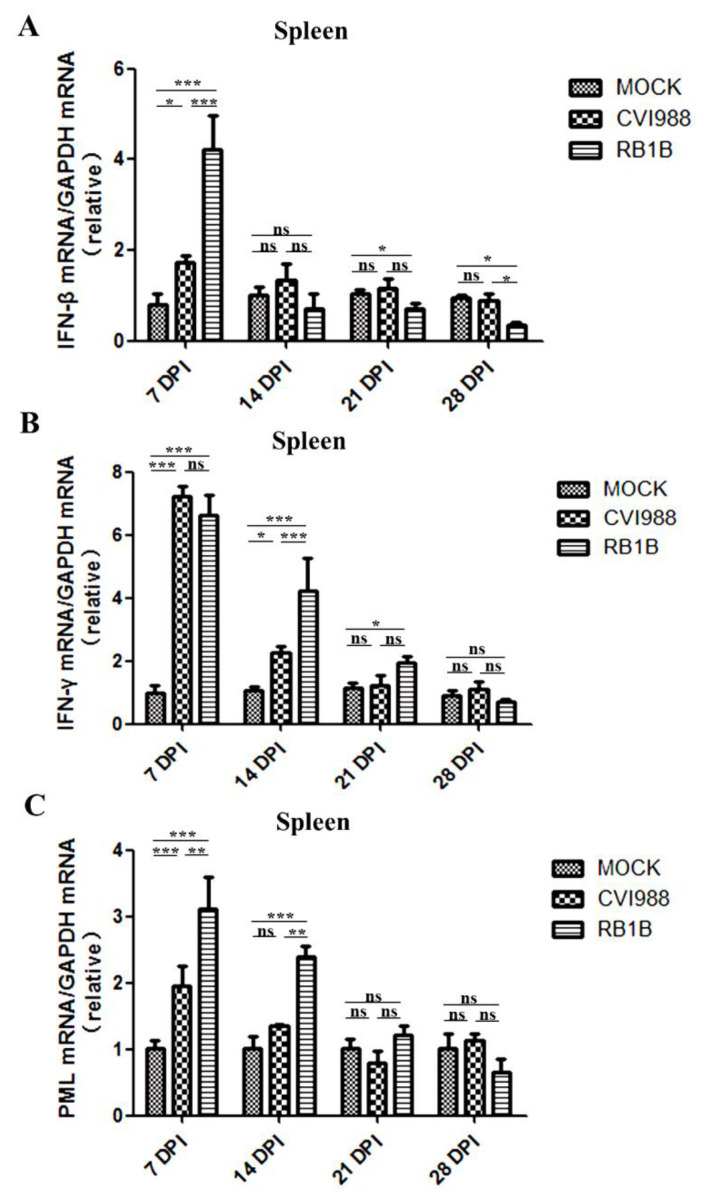
IFN-β, IFN-γ, and PML transcripts in the spleen inoculated with MDV/CVI988 and MDV/RB1B. (**A**): Specific IFN-β transcripts in the spleen; (**B**): specific IFN-γ transcripts in the spleen; (**C**): specific PML transcripts in the spleen. The statistical results are shown as the means ±SD of each experimental group. Statistical significance between each two experimental groups (CVI988 vs. MOCK, RB1B vs. MOCK, CVI988 vs. RB1B) was assessed by *t*-test (* *p* < 0.05; ** *p* < 0.01; *** *p* < 0.001; ns, no significance).

**Figure 3:**
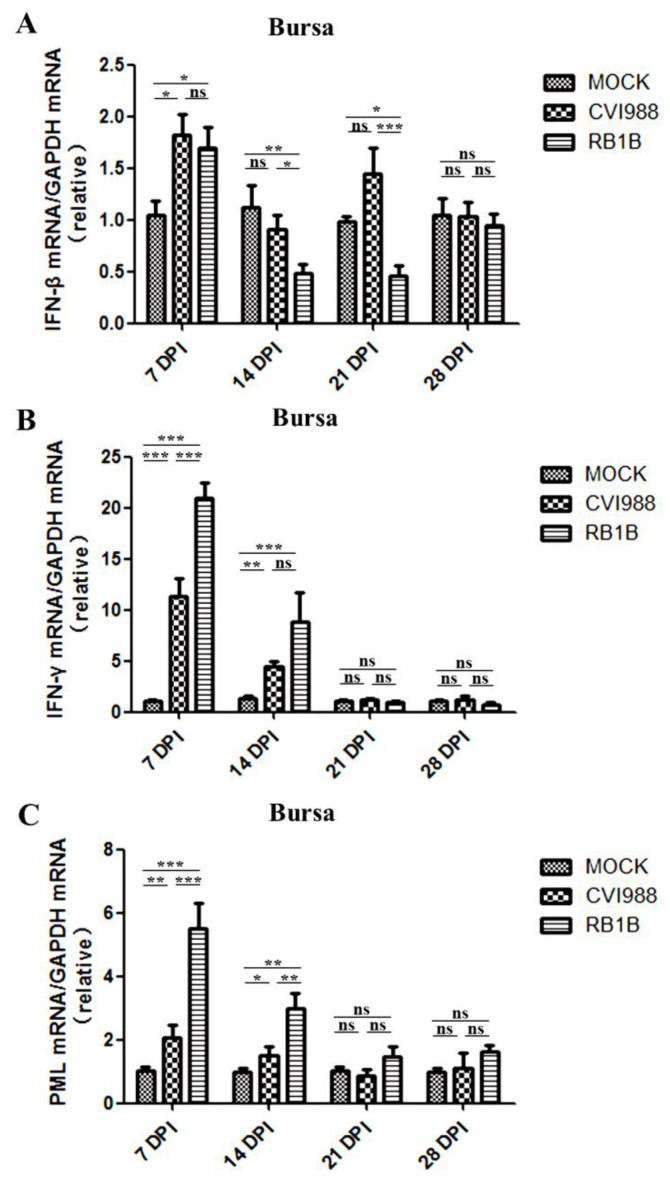
IFN-β, IFN-γ, and PML transcripts in the bursa inoculated with MDV/CVI988 and MDV/RB1B. (**A**): Specific IFN-β transcripts in the bursa; (**B)**: specific IFN-γ transcripts in the bursa; (**C**): specific PML transcripts in the bursa. The statistical results are shown as the means ± SD of each experimental group. Statistical significance between each two experimental groups (CVI988 vs. MOCK, RB1B vs. MOCK, CVI988 vs. RB1B) was assessed by *t*-test (* *p* < 0.05; ** *p* < 0.01; *** *p* < 0.001; ns, no significance).

To determine the interferon productions induced by MDV infection in the spleen and bursa, IFN-β, IFN-γ, and PML mRNA levels were analyzed using RT-qPCR assays. The level of IFN-β mRNA was significantly upregulated in both the spleen and bursa of chickens infected with MDV/CVI988 and MDV/RB1B in the early stage of infection (7 dpi), but it was inhibited to different degrees thereafter in the MDV/RB1B experimental group (Figure 2 and Figure 3). Compared with MDV/CVI988, MDV/RB1B infection induced more significant transcriptional upregulation of IFN-β in the spleen at 7 dpi. However, at 14, 21 and 28 dpi, MDV/RB1B infection resulted in more severe suppression of IFN-β expression in the spleen than that of MDV/CVI988 infection (Figure 2A). Unlike IFN-β, the changes of IFN-γ transcription in the spleen were significantly upregulated by MDV/RB1B or MDV/CVI988 infections. The upregulation of IFN-γ triggered by MDV/RB1B infection persisted until 21 dpi in the spleen, whereas that triggered by MDV/CVI988 infection only lasted until 14 dpi (Figure 2B). This suggests that MDV/RB1B infection induces a stronger interferon type II response in the spleen than MDV/CVI988. As the core component of PML-NBs, PML is an important host-defense molecule that inhibits early herpes virus infection and favors the establishment of latent infection. It is also an interferon stimulator positively regulated by IFN-γ. The results of our investigation showed that the transcription levels of PML in the spleen were upregulated at 7 and 14 dpi after MDV/RB1B infection, but for CVI988 infection, they were only upregulated at 7 dpi. Moreover, there was no significant difference in PML mRNA levels in the spleens of the two MDV-infected groups and the control group at 21 dpi (Figure 2C).

IFN-β transcription in the bursa of chickens infected with MDV/CVI988 or MDV/RB1B was significantly upregulated at 7 dpi. However, in contrast to the results detected in spleens, there was no significant difference in the upregulation of IFN-β mRNA in the bursa induced by infection with these two MDV strains at 7 dpi. MDV/RB1B infection induced a more effective suppression of IFN-β expression than MDV/CVI988 in the bursa at 14–21 dpi (Figure 3A). The IFN-γ transcription levels in the bursa of chickens infected with MDV/RB1B or MDV/CVI988 increased significantly at 7 and 14 dpi. Consistent with the results in the spleen, MDV/RB1B infection also induced more vigorous IFN-γ transcriptional activation in the bursa compared with MDV/CVI988 (Figure 3B). After MDV infection, the changes of PML transcription in the bursa also occurred at 7 and 14 dpi. MDV/RB1B infection resulted in a six-fold increase in PML mRNA levels at 7 dpi and a three-fold increase at 14 dpi, whereas PML mRNA levels in the bursa upregulated by MDV/CVI988 infection less than two-fold at 7 and 14 dpi (Figure 3C).

### 3.3. Effects of MDV Replication on and Chemokine Expression in the Spleen and Bursa

**Figure 4:**
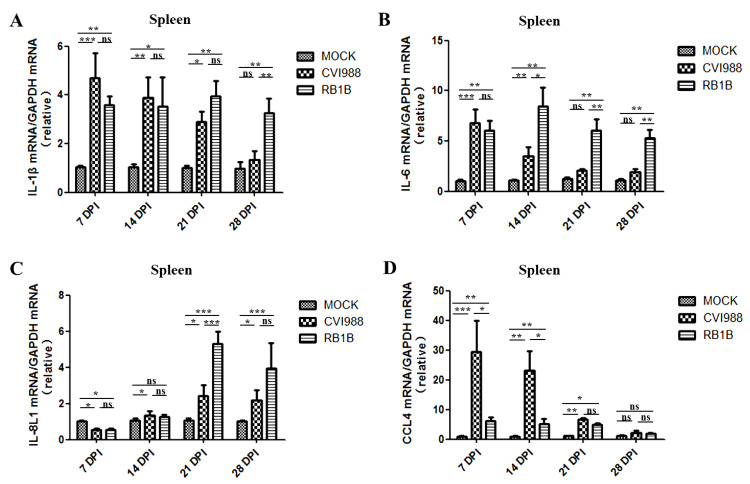
IL-1β, IL-6, IL-8L1, andCCL4 transcripts in the spleen inoculated with MDV/CVI988 and MDV/RB1B. (**A**): Specific IL-1β transcripts in the spleen; (**B**): specific IL-6 transcripts in the spleen; (**C**): specific IL-8L1 transcripts in the spleen; (**D**): specific CCL4 transcripts in the spleen. The statistical results are shown as the means ± SD of each experimental group. Statistical significance between each two experimental groups (CVI988 vs. MOCK, RB1B vs. MOCK, CVI988 vs. RB1B) was assessed by *t*-test (* *p* < 0.05; ** *p* < 0.01; *** *p* < 0.001; ns, no significance).

Chickens infected with MDV/RB1B or MDV/CVI988 had significantly increased mRNA levels of IL-1β in the spleen compared with the mock chickens at 7, 14, 21, and 28 dpi. The mRNA levels of IL-1β induced by MDV/RB1B and MDV/CVI988 were not significantly different at 7, 14, and 21dpi, while MDV/RB1B induced higher IL-1β expression at 28 dpi (Figure 4A). MDV/CVI988 infection increased IL-6 mRNA levels in chicken spleens by up to six-fold at 7 dpi and four-fold at 14 dpi. Nonetheless, it had no obvious effect on IL-6 expression at 21 and 28 dpi in the spleen. The IL-6 mRNA levels in chickens infected with MDV/RB1B increased at all four time points post-inoculation, ranging from five-fold at 7 dpi to almost ten-fold at 28 dpi. In addition, the transcriptional activation of IL-6 in the spleen, induced by MDV/RB1B, was higher than that induced by MDV/CVI988 at 14, 21, and 28 dpi (Figure 4B). The transcription of IL-8L1 in the spleen was inhibited by MDV/RB1B or MDV/CVI988 infection in chickens at 7 dpi but was upregulated thereafter, particularly MDV/RB1B infection at 21 and 28 dpi (Figure 4C). MDV/CVI988 infection resulted in a substantial upregulation of CCL4 mRNA in the spleen of chickens, up to thirty-fold compared with uninfected chickens at 7 and 14 dpi. Although MDV/RB1B infection also positively regulated CCL4 transcription, the extent of upregulation was significantly less than that of MDV/CVI988 at 7 and 14 dpi. At 21 dpi, both MDV/RB1B and MDV/CVI988 increased the mRNA levels of CCL4 in the spleen, and there was no significant difference between the two MDV strains (Figure 4D).

**Figure 5:**
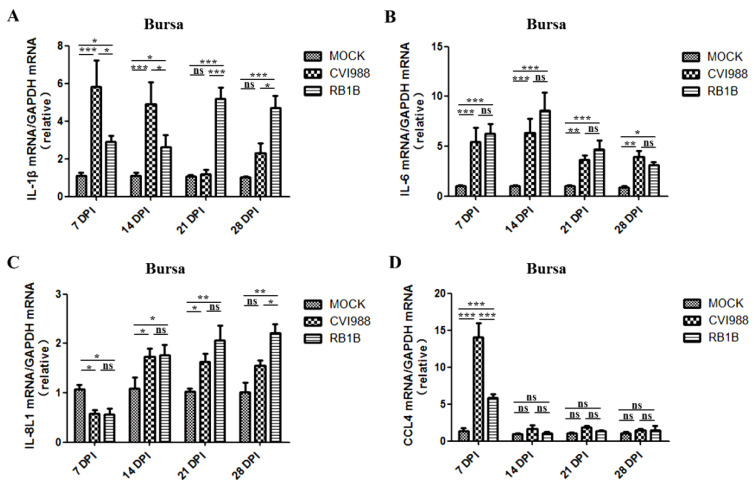
IL-1β, IL-6, IL-8L1, andCCL4 transcripts in the bursa inoculated with MDV/CVI988 and MDV/RB1B. (**A**) Specific IL-1β transcripts in the bursa; (**B**) specific IL-6 transcripts in the bursa; (**C**) specific IL-8L1 transcripts in the bursa; (**D**) specific CCL4 transcripts in the bursa. The statistical results are shown as the means ± SD of each experimental group. Statistical significance between each two experimental groups (CVI988 vs. MOCK, RB1B vs. MOCK, CVI988 vs. RB1B) was assessed by *t*-test (* *p* < 0.05; ** *p* < 0.01; *** *p* < 0.001; ns, no significance).

Likewise, chickens infected with MDV/CVI988 at 7 and 14 dpi showed significantly increased levels of IL-1β in the bursa compared with the uninfected control chickens, but the MDV/RB1B infection-induced upregulation of IL-1β transcription lasted from 7 to 28 dpi (Figure 5A). The mRNA levels of IL-6 in the bursa were significantly upregulated at 7, 14, 21, and 28 dpi by either MDV/RB1B or MDV/CVI988 infection, and there was no significant difference between the two strains, which was inconsistent with the results in the spleen (Figure 5B). The transcription of IL-8L1 in the bursa of MDV/CVI988- or MDV/RB1B-infected chickens were both inhibited at 7 dpi, but then, the transcription levels began to increase. The mRNA level of IL-8L1 in the bursa of the MDV/CVI infection group was higher than that in the control group at 14, 21, and 28 dpi. In the MDV/RB1B infection group, the IL-8L1 transcription level was higher than that in the control group at 21 and 28 dpi (Figure 5C). The bursa CCL4 mRNA levels in chickens infected with MDV/CVI988 increased by 14-fold compared with those in the control group at 7 dpi and were significantly higher than that of the MDV/RB1B-infected group. However, there was no significant difference among all experimental groups at 14, 21, and 28 dpi (Figure 5D).

## 4. Discussion

As an oncogenic herpes virus, the infection of MDV comprises the early cytolytic phase, latency, reactivation, and transformation phases [17]. The initial cytolysis phase after MDV infection occurs around 2–7 dpi when B lymphocytes in the spleen and bursa are the main target cells of MDV proliferation [18]. Then, MDV infection enters the latency phase at around 7–10 dpi; the main target cells in this infection phase are CD4+ T cells [19,20]. The late reactivation and immunosuppressive phases are also initiated in CD4+ T cells at approximately 18 dpi, and the final transformation phase is characterized by multiple organ tumors originating from CD4+ T cell lymphoma at approximately 28 dpi [21]. Especially in susceptible chickens, a second wave of productive infection and cytolysis begins 14–21 dpi after MDV infection, which is known as the reactivation or late cytolytic phase [21]. MDV infections result in alterations in the infiltration and proliferation of B cells and CD4+ T cells. As main lymphoid organs in chickens, both the spleen and bursa not only support MDV replication, but also play an important role in immune response [15,22,23]. Moreover, the spleen mainly contains B and T lymphocytes, which are dominant target cells of MDV. As the main site of B lymphocyte maturation and development, the bursa is the main target organ during the early stages of MDV infection. Moreover, the virus continues to replicate in the bursa within 5 weeks of MDV infection, causing varying degrees of bursa atrophy depending on the strain [24].

In this study, we examined the viral loads of MDV vaccine strain MDV/CVI988 and vv strain MDV/RB1B as a measure of the kinetics of replication, as well as interferon and inflammatory cytokines, in the spleen and bursa of chickens. The MDV viral load in the spleen and bursa show that MDV/RB1B has a significantly stronger replication ability than MDV/CVI988. Earlier studies by our group showed that MDV/RB1B replication was significantly stronger than that of CVI988 in the bursa and spleen at 5 dpi [15]. Combined with the results of this study, the replication ability of MDV/RB1B was also significantly stronger than that of MDV/CVI988 in the latent and reactivation infection phases (7–28 dpi) in vivo. We speculate that MDV strains with stronger virulence are more capable of replicating in chickens, which is consistent with the results of Jarosinski et al. showing that the vv+ MDV strain, RK-1, is significantly more capable of replicating in vivo than the virulent strain, JM-16, within 14 days after infection [25]. The bursa plays an immune function in chickens until 60 days of age and undergoes gradual involution after 14 days of age, whereas non-bursal tissue gradually take over its role [26]. Our results showed that viral loads in the spleen after infection with the MDV vaccine strain and vvMDV strain were higher than those in the bursa, which was consistent with the results of Baigent et al. on MDV/CVI988 replication in vivo [27]. This may be because lymphoid organs are the dominant sites for MDV replication and the spleen is the peripheral lymphoid organ that aggregates more T cells than the bursa, facilitating MDV reactivation from latent infection [28]. Therefore, we hypothesized that the higher replication level of MDV/RB1B in the spleen at 21 and 28 dpi than that of MDV/CVI988 might be related to more transformed T cells in the spleen, which support the productive replication of MDV. It has been demonstrated that the respective specific cleavage patterns of MDV/CVI988 and MDV/RB1B after infecting host cells are important factors affecting viral pathogenicity and replication [29,30]. We present that the differential replication between MDV/CVI988 and MDV/RB1B in spleens and bursas may be related to the strain-specific splicing mode of MDV in different cells. In addition, due to the different microenvironment of tissues, the replication ability of MDV varies in each tissue, which agrees with the result in the study of Haq et al. that MDV/RB1B replication ability is stronger in the feather follicle than in the spleen [31].

Although type I interferons did not prevent multiorgan tumors caused by vMDV infection, they were shown to inhibit MDV replication and alleviate clinical symptoms [32]. In our study, the expression of IFN-β at 7 dpi in the spleen and bursa of chickens infected with MDV/CVI988 and MDV/RB1B increased; however, different extents of expression inhibition were observed at 14, 21, and 28 dpi, and the downregulation of IFN-β transcription caused by the MDV/RB1B strain was more obvious. Once the pathogen was detected, pattern recognition receptors initiated the innate immune response and then triggered the production of type I interferon [33]. Therefore, the upregulation of IFN-β transcription at 7 dpi is an inevitable result of the innate immune response activation caused by virus proliferation. However, it has been reported that MDV inhibits the innate immune response by restraining the cGAS-STING DNA-sensing pathway in vitro and in vivo [34]. As the viral load of MDV is relatively low in the early infection phase, the host’s innate immune response is dominant. With the increase in viral load and viral protein expression, the type I IFN response of the host no longer dominates, and IFN-β transcription is inhibited from 14 dpi. The inhibitory effects of MDV/RB1B on IFN-β were stronger than those of MDV/CVI988, likely due to the higher RB1B viral load in the spleen and bursa, which may also be caused by the higher virulence of RB1B to immune cells, resulting in impaired immune responses by virus-induced apoptosis [35].

IFN-γ is thought to play a crucial role in the anti-MDV immune response and can directly or indirectly resist MDV replication through the induction of nitric oxide [36,37,38]. The results of the study indicated that the expression of IFN-γ in the two lymphoid organs was upregulated by MDV infection, and the vv strain MDV/RB1B induced stronger IFN-γ transcriptional upregulation than MDV/CVI988 at 7, 14, and 21 dpi, which showed a trend similar to that reported by Bavananthasivam et al. [39]. As previous studies have reported that MDV activates NK cells through Meq, which in turn upregulates IFN-γ transcription, this may be due to the higher MDV/RB1B replication in the spleen and bursa inducing a greater number of activated NK cells and T-cells [40]. It is coincident with the evidence that the mRNA level of IFN-γ is significantly increased at 5 and 7 dpi following RB1B strain MDV infection [15,41]. Furthermore, we found that PML expression was up-regulated by both MDV strains prior to 14 dpi, and MDV/RB1B infection showed a higher efficiency than that of MDV/CVI988 infection (Figure 2C and Figure 3C). As a core component of the PML nucleosome, PML plays a major role in facilitating the establishment of a latent state for herpes viruses such as HSV-1 [42,43]. PML expression is positively regulated by IFN-γ and has been shown to inhibit herpes virus DNA replication and transcription [44,45]. Moreover, PML plays a major role in composing PML-NBs, which favor the establishment of a latent state for herpes viruses such as HSV-1 through a PML NB/Histone H3.3/H3.3 Chaperone Axis [42,46]. Considering the role of PML in the process of latent infection with the herpes virus, these early high levels of IFN-γ and PML expression in chickens infected with MDV/RB1B could drive latent infection to occur earlier [47]. These findings suggest that, unlike MDV/CVI988 immunization, MDV/RB1B infection might prolong the latent infection, leading to CD4+ T cell transformation.

IL-1β, IL-6, IL8L1, and CCL4 are associated with B cell activation as well as antigenic signal transduction to T cells [48,49,50]. IL-1β is an important pro-inflammatory cytokine indispensable for tissue repair and cellular defense [51,52]. The transcriptional upregulation of IL-1β is thought to be related to the occurrence of tumors, including lung, colon, and breast cancers, and it is also related with poorer prognosis [53]. We found that IL-1β was highly expressed in the spleen and bursa of chickens at 7 and 14 after MDV/CVI988 infection (Figure 4A and Figure 5A), and the expression level gradually decreased with time, which was consistent with the data reported previously on N2a strain chickens infected with the MDV JM-6 strain [25]. However, infection with the MDV/RB1B strain resulted in persistently high levels of IL-1β transcription in both the spleen and bursa. It has been confirmed that the up-regulation of IL-1β is positively associated with morbidity caused by HSV-1 infection [54]. Therefore, we speculated that this phenomenon of increased viral load and a continuous transcriptional up-regulation of IL-1β caused by MDV/RB1B infection may be related to its pathological damage to lymphoid organs and CD4+ T cell transformation due to viral replication, but the specific mechanism requires further study. Pro-inflammatory cytokine IL-6 is elevated in susceptible chickens during MDV infections [55]. Mohamed et al. also found that the expression of IL-6 in the spleen of RB1B-infected unvaccinated chickens was higher than that in vaccine-immunized chickens [56]. Combined with our experimental findings that MDV/RB1B infection induced a higher level of IL-6 mRNA in the spleen at 14, 21, and 28 dpi than MDV/CVI988, we speculated that the persistently high levels of IL-6 expression in lymphoid organs might be a differential marker between vvMDV strain infection and MDV/CVI988 immunization. The transcriptional expression of IL-6 and IL-8 is rapidly induced by many herpes virus infections, including HSV-1, HCMV, and VZV, which represent the activation of pro-inflammatory cytokines [57,58,59]. However, IL-8L1 is only encoded in chickens and is believed to function as an IL-8. In addition, it may have similar functions to MDV viral IL8 (vIL-8) in the hinge of infection from B to T lymphocytes, which promotes lymphoma formation through the subsequent recruitment of CD4+ T cells [60]. The IL-8L1 mRNA level of the spleen and bursa induced by MDV/RB1B was higher than that induced by MDV/CVI988 at 21 and 28 dpi (Figure 4C and Figure 5C). This implies that MDV/RB1B infection initiates a more dramatic inflammatory response compared to MDV/CVI988 immunization. CCL4, highly expressed by effector CD8+ T cells, has been proven to play a crucial role in immune response, cellular recruitment, and direct anti-viral activity against HSV-1 [61]. Our results demonstrated that vaccine strain MDV/CVI988 infection resulted in high levels of CCL4 expression in the key lymphoid organs of the chicken, particularly in the bursa infected at 7 dpi and the spleen within 21 days after infection (Figure 4D and Figure 5D). Considering the positive role of CCL4 in the suppression of herpes virus infection, we speculated that the up-regulation of CCL4 expression after MDV/CVI988 vaccination may be the outcome of the CD8+ CTL response, and it is expected to play a significant role in the defense against vvMDV infection [62].

## 5. Conclusions

In this study, we found that the replication ability of very virulent MDV/RB1B was significantly stronger than that of MDV/CVI988 within 28 days post-infection, and the replication levels of both MDV strains in the spleen were significantly higher than those in the bursa. During the latent and reactivation phase of MDV infection (7–28 dpi), the transcriptional upregulation of chicken IL-1β, IL6, IL-8L1, IFN-γ, and PML in the spleen and bursa induced by MDV/RB1B infection was overall stronger than that of MDV/CVI988. However, compared to MDV/RB1B infection, MDV/CVI988 infection resulted in a more effective transcriptional activation of CCL4 in the latent infection phase (7–14 dpi), which may be a characteristic distinguishing the vaccine MDV strain from the vvMDV strain. This study provides new insights with which to explore the mechanisms of MDV immunity and pathogenesis.

## Figures and Tables

**Table 1:** Primers for qPCR in this study.

Name	Sequence (5′-3′)	Name	Sequence (5′-3′)
Meq-F	CCCAACAGCCCCTCCAAACAC	Meq-R	CTTCATGGAGTTTGTCTACA
ChIFN-β-F	CCTCAACCAGATCCAGCATTAC	ChIFN-β-R	CCCAGGTACAAGCACTGTAGTT
ChIFN-γ-F	GCCGCACATCAAACACATATCTG	ChIFN-γ-R	GCGCTGGATTCTCAAGTCGTTC
ChPML-F	TCTACCGGCGCATTGTCAG	ChPML-R	TGGGTCTCGAAGCACTTTGT
IL-1β-F	CCCGCTTCATCTTCTACCGC	IL-1β-R	GCTTGTAGGTGGCGATGTTG
IL-6-F	AAATCCCTCCTCGCCAATCT	IL-6-R	CCCTCACGGTCTTCTCCATAAA
IL8L1-F	CCTTCAGCTTTGTGGCAGAC	IL8L1-R	GGAGGAGGTAGGACGTTTTTG
CCL4-F	CCAGAATGCTGGTAATCGACG	CCL4-R	GGTGATGAACACAACACCAGC
GAPDH-F	AGAACATCATCCCAGCGT	GAPDH-R	AGCCTTCACTACCCTCTTG

* F: Forward primer; R: Reverse primer.

## Data Availability

The data presented in this study are available on request from the corresponding author. The data are not publicly available due to data are still being processed to produce other papers.

## References

[1] Nair V. (2018). Spotlight on avian pathology: Marek’s disease. Avian Pathol. J. W.V.P.A.

[2] Song B., Zeb J., Hussain S., Aziz M.U., Circella E., Casalino G., Camarda A., Yang G., Buchon N., Sparagano O. (2022). A Review on the Marek’s Disease Outbreak and Its Virulence-Related meq Genovariation in Asia between 2011 and 2021. Animals.

[3] Osterrieder N., Kamil J.P., Schumacher D., Tischer B.K., Trapp S. (2006). Marek’s disease virus: From miasma to model. Nat. Rev. Microbiol..

[4] Abdul-Careem M.F., Javaheri-Vayeghan A., Shanmuganathan S., Haghighi H.R., Read L.R., Haq K., Hunter D.B., Schat K.A., Heidari M., Sharif S. (2009). Establishment of an aerosol-based Marek’s disease virus infection model. Avian Dis..

[5] Boodhoo N., Gurung A., Sharif S., Behboudi S. (2016). Marek’s disease in chickens: A review with focus on immunology. Vet. Res..

[6] Robinson C.M., Cheng H.H., Delany M.E. (2014). Temporal kinetics of Marek’s disease herpesvirus: Integration occurs early after infection in both B and T cells. Cytogenet. Genome Res..

[7] Teng M., Zheng L.P., Li H.Z., Ma S.M., Zhu Z.J., Chai S.J., Yao Y., Nair V., Zhang G.P., Luo J. (2022). Pathogenicity and Pathotype Analysis of Henan Isolates of Marek’s Disease Virus Reveal Long-Term Circulation of Highly Virulent MDV Variant in China. Viruses.

[8] Biggs P.M., Nair V. (2012). The long view: 40 years of Marek’s disease research and Avian Pathology. Avian Pathol. J. W.V.P.A.

[9] Baigent S.J., Nair V.K., Le Galludec H. (2016). Real-time PCR for differential quantification of CVI988 vaccine virus and virulent strains of Marek’s disease virus. J. Virol. Methods.

[10] Petherbridge L., Howes K., Baigent S.J., Sacco M.A., Evans S., Osterrieder N., Nair V. (2003). Replication-competent bacterial artificial chromosomes of Marek’s disease virus: Novel tools for generation of molecularly defined herpesvirus vaccines. J. Virol..

[11] Boodhoo N., Behboudi S. (2021). Differential Virus-Specific IFN-Gamma Producing T Cell Responses to Marek’s Disease Virus in Chickens With B19 and B21 MHC Haplotypes. Front. Immunol..

[12] Bacon L.D., Witter R.L. (1993). Influence of B-haplotype on the relative efficacy of Marek’s disease vaccines of different serotypes. Avian Dis..

[13] Conradie A.M., Bertzbach L.D., Trimpert J., Patria J.N., Murata S., Parcells M.S., Kaufer B.B. (2020). Distinct polymorphisms in a single herpesvirus gene are capable of enhancing virulence and mediating vaccinal resistance. PLoS Pathog..

[14] Read A.F., Baigent S.J., Powers C., Kgosana L.B., Blackwell L., Smith L.P., Kennedy D.A., Walkden-Brown S.W., Nair V.K. (2015). Imperfect Vaccination Can Enhance the Transmission of Highly Virulent Pathogens. PLoS Biol..

[15] Jin H., Kong Z., Mehboob A., Jiang B., Xu J., Cai Y., Liu W., Hong J., Li Y. (2020). Transcriptional Profiles Associated with Marek’s Disease Virus in Bursa and Spleen Lymphocytes Reveal Contrasting Immune Responses during Early Cytolytic Infection. Viruses.

[16] Spatz S.J., Petherbridge L., Zhao Y., Nair V. (2007). Comparative full-length sequence analysis of oncogenic and vaccine (Rispens) strains of Marek’s disease virus. J. Gen. Virol..

[17] Berthault C., Larcher T., Hartle S., Vautherot J.F., Trapp-Fragnet L., Denesvre C. (2018). Atrophy of primary lymphoid organs induced by Marek’s disease virus during early infection is associated with increased apoptosis, inhibition of cell proliferation and a severe B-lymphopenia. Vet. Res..

[18] Baaten B.J., Staines K.A., Smith L.P., Skinner H., Davison T.F., Butter C. (2009). Early replication in pulmonary B cells after infection with Marek’s disease herpesvirus by the respiratory route. Viral Immunol..

[19] Bavananthasivam J., Alkie T.N., Astill J., Abdul-Careem M.F., Wootton S.K., Behboudi S., Yitbarek A., Sharif S. (2018). In ovo administration of Toll-like receptor ligands encapsulated in PLGA nanoparticles impede tumor development in chickens infected with Marek’s disease virus. Vaccine.

[20] Abdul-Careem M.F., Read L.R., Parvizi P., Thanthrige-Don N., Sharif S. (2009). Marek’s disease virus-induced expression of cytokine genes in feathers of genetically defined chickens. Dev. Comp. Immunol..

[21] Calnek B.W. (1986). Marek’s disease—A model for herpesvirus oncology. Crit. Rev. Microbiol..

[22] Zhang Q., Sun X., Wang T., Chen B., Huang Y., Chen H., Chen Q. (2019). The Postembryonic Development of the Immunological Barrier in the Chicken Spleens. J. Immunol. Res..

[23] Ifrah M.E., Perelman B., Finger A., Uni Z. (2017). The role of the bursa of Fabricius in the immune response to vaccinal antigens and the development of immune tolerance in chicks (*Gallus domesticus*) vaccinated at a very young age. Poultry science.

[24] Islam A.F., Wong C.W., Walkden-Brown S.W., Colditz I.G., Arzey K.E., Groves P.J. (2002). Immunosuppressive effects of Marek’s disease virus (MDV) and herpesvirus of turkeys (HVT) in broiler chickens and the protective effect of HVT vaccination against MDV challenge. Avian Pathol. J. W.V.P.A.

[25] Jarosinski K.W., Njaa B.L., O’Connell P.H., Schat K.A. (2005). Pro-inflammatory responses in chicken spleen and brain tissues after infection with very virulent plus Marek’s disease virus. Viral Immunol..

[26] Paramithiotis E., Ratcliffe M.J. (1993). Bursa-dependent subpopulations of peripheral B lymphocytes in chicken blood. Eur. J. Immunol..

[27] Baigent S.J., Smith L.P., Currie R.J.W., Nair V.K. (2005). Replication kinetics of Marek’s disease vaccine virus in feathers and lymphoid tissues using PCR and virus isolation. J. Gen. Virol..

[28] Calnek B.W. (2001). Pathogenesis of Marek’s disease virus infection. Curr. Top. Microbiol. Immunol..

[29] Sadigh Y., Tahiri-Alaoui A., Spatz S., Nair V., Ribeca P. (2020). Pervasive Differential Splicing in Marek’s Disease Virus can Discriminate CVI-988 Vaccine Strain from RB-1B Very Virulent Strain in Chicken Embryonic Fibroblasts. Viruses.

[30] Bertzbach L.D., Pfaff F., Pauker V.I., Kheimar A.M., Hoper D., Hartle S., Karger A., Kaufer B.B. (2019). The Transcriptional Landscape of Marek’s Disease Virus in Primary Chicken B Cells Reveals Novel Splice Variants and Genes. Viruses.

[31] Haq K., Fear T., Ibraheem A., Abdul-Careem M.F., Sharif S. (2012). Influence of vaccination with CVI988/Rispens on load and replication of a very virulent Marek’s disease virus strain in feathers of chickens. Avian Pathol. J. W.V.P.A.

[32] Bertzbach L.D., Harlin O., Hartle S., Fehler F., Vychodil T., Kaufer B.B., Kaspers B. (2019). IFNalpha and IFNgamma Impede Marek’s Disease Progression. Viruses.

[33] Brubaker S.W., Bonham K.S., Zanoni I., Kagan J.C. (2015). Innate immune pattern recognition: A cell biological perspective. Annu. Rev. Immunol..

[34] Li K., Liu Y., Xu Z., Zhang Y., Luo D., Gao Y., Qian Y., Bao C., Liu C., Zhang Y. (2019). Avian oncogenic herpesvirus antagonizes the cGAS-STING DNA-sensing pathway to mediate immune evasion. PLoS Pathog..

[35] Gurung A., Kamble N., Kaufer B.B., Pathan A., Behboudi S. (2017). Association of Marek’s Disease induced immunosuppression with activation of a novel regulatory T cells in chickens. PLoS Pathog..

[36] Xing Z., Schat K.A. (2000). Inhibitory effects of nitric oxide and gamma interferon on in vitro and in vivo replication of Marek’s disease virus. J. Virol..

[37] Jiang B., Wang J., Liu W., Cheng J., Xu J., Cao M., Li Y. (2022). Comparative transcriptome analysis of MDBK cells reveals that BoIFN-gamma augmented host immune responses to bovine herpesvirus 1 infection. Front. Microbiol..

[38] Jud A., Kotur M., Berger C., Gysin C., Nadal D., Lunemann A. (2017). Tonsillar CD56brightNKG2A+ NK cells restrict primary Epstein-Barr virus infection in B cells via IFN-gamma. Oncotarget.

[39] Bavananthasivam J., Astill J., Matsuyama-Kato A., Taha-Abdelaziz K., Shojadoost B., Sharif S. (2021). Gut microbiota is associated with protection against Marek’s disease virus infection in chickens. Virology.

[40] Bertzbach L.D., van Haarlem D.A., Hartle S., Kaufer B.B., Jansen C.A. (2019). Marek’s Disease Virus Infection of Natural Killer Cells. Microorganisms.

[41] Djeraba A., Musset E., Bernardet N., Le Vern Y., Quere P. (2002). Similar pattern of iNOS expression, NO production and cytokine response in genetic and vaccination-acquired resistance to Marek’s disease. Vet. Immunol. Immunopathol..

[42] Cohen C., Corpet A., Roubille S., Maroui M.A., Poccardi N., Rousseau A., Kleijwegt C., Binda O., Texier P., Sawtell N. (2018). Promyelocytic leukemia (PML) nuclear bodies (NBs) induce latent/quiescent HSV-1 genomes chromatinization through a PML NB/Histone H3.3/H3.3 Chaperone Axis. PLoS Pathog..

[43] Rai T.S., Glass M., Cole J.J., Rather M.I., Marsden M., Neilson M., Brock C., Humphreys I.R., Everett R.D., Adams P.D. (2017). Histone chaperone HIRA deposits histone H3.3 onto foreign viral DNA and contributes to anti-viral intrinsic immunity. Nucleic Acids Res..

[44] Chang H.R., Munkhjargal A., Kim M.J., Park S.Y., Jung E., Ryu J.H., Yang Y., Lim J.S., Kim Y. (2018). The functional roles of PML nuclear bodies in genome maintenance. Mutat. Res..

[45] Gu H., Zheng Y. (2016). Role of ND10 nuclear bodies in the chromatin repression of HSV-1. Virol. J..

[46] Catez F., Picard C., Held K., Gross S., Rousseau A., Theil D., Sawtell N., Labetoulle M., Lomonte P. (2012). HSV-1 genome subnuclear positioning and associations with host-cell PML-NBs and centromeres regulate LAT locus transcription during latency in neurons. PLoS Pathog..

[47] Scherer M., Stamminger T. (2016). Emerging Role of PML Nuclear Bodies in Innate Immune Signaling. J. Virol..

[48] Chan D., Bennett P.R., Lee Y.S., Kundu S., Teoh T.G., Adan M., Ahmed S., Brown R.G., David A.L., Lewis H.V. (2022). Microbial-driven preterm labour involves crosstalk between the innate and adaptive immune response. Nat. Commun..

[49] Cardoso Dal Pont G., Lee A., Bortoluzzi C., Farnell Y.Z., Gougoulias C., Kogut M.H. (2022). Novel model for chronic intestinal inflammation in chickens: (2) Immunologic mechanism behind the inflammatory response. Dev. Comp. Immunol..

[50] Schilling M.A., Katani R., Memari S., Cavanaugh M., Buza J., Radzio-Basu J., Mpenda F.N., Deist M.S., Lamont S.J., Kapur V. (2018). Transcriptional Innate Immune Response of the Developing Chicken Embryo to Newcastle Disease Virus Infection. Front. Genet..

[51] Ni Gabhann-Dromgoole J., de Chaumont C., Shahnazaryan D., Smith S., Malone C., Hassan J., De Gascun C.F., Jefferies C.A., Murphy C.C. (2019). Systemic IL-1beta production as a consequence of corneal HSV-1 infection-contribution to the development of herpes simplex keratitis. Int. J. Ophthalmol..

[52] Dinarello C.A. (2018). Overview of the IL-1 family in innate inflammation and acquired immunity. Immunol. Rev..

[53] Rebe C., Ghiringhelli F. (2020). Interleukin-1beta and Cancer. Cancers.

[54] Karaba A.H., Figueroa A., Massaccesi G., Botto S., DeFilippis V.R., Cox A.L. (2020). Herpes simplex virus type 1 inflammasome activation in proinflammatory human macrophages is dependent on NLRP3, ASC, and caspase-1. PLoS ONE.

[55] Kaiser P., Underwood G., Davison F. (2003). Differential cytokine responses following Marek’s disease virus infection of chickens differing in resistance to Marek’s disease. J. Virol..

[56] Abdul-Careem M.F., Hunter B.D., Parvizi P., Haghighi H.R., Thanthrige-Don N., Sharif S. (2007). Cytokine gene expression patterns associated with immunization against Marek’s disease in chickens. Vaccine.

[57] Jones D., Neff C.P., Palmer B.E., Stenmark K., Nagel M.A. (2017). Varicella zoster virus-infected cerebrovascular cells produce a proinflammatory environment. Neurol. Neuroimmunol. Neuroinflamm..

[58] Costa H., Nascimento R., Sinclair J., Parkhouse R.M. (2013). Human cytomegalovirus gene UL76 induces IL-8 expression through activation of the DNA damage response. PLoS Pathog..

[59] Li H., Zhang J., Kumar A., Zheng M., Atherton S.S., Yu F.S. (2006). Herpes simplex virus 1 infection induces the expression of proinflammatory cytokines, interferons and TLR7 in human corneal epithelial cells. Immunology.

[60] Engel A.T., Selvaraj R.K., Kamil J.P., Osterrieder N., Kaufer B.B. (2012). Marek’s disease viral interleukin-8 promotes lymphoma formation through targeted recruitment of B cells and CD4+ CD25+ T cells. J. Virol..

[61] Nakayama T., Shirane J., Hieshima K., Shibano M., Watanabe M., Jin Z., Nagakubo D., Saito T., Shimomura Y., Yoshie O. (2006). Novel antiviral activity of chemokines. Virology.

[62] Bystry R.S., Aluvihare V., Welch K.A., Kallikourdis M., Betz A.G. (2001). B cells and professional APCs recruit regulatory T cells via CCL4. Nat. Immunol..

